# Evaluation of *Pseudomonas fulva* PS9.1 and *Bacillus velezensis* NWUMFkBS10.5 as Candidate Plant Growth Promoters during Maize-*Fusarium* Interaction

**DOI:** 10.3390/plants11030324

**Published:** 2022-01-26

**Authors:** Adetomiwa A. Adeniji, Olubukola O. Babalola

**Affiliations:** 1 Human Metabolomics, Faculty of Natural and Agricultural Science, Private Bag X6001, Box 269, Potchefstroom 2531, South Africa; 2 Food Security and Safety Niche Area, Faculty of Natural and Agricultural Science, North-West University, Private Bag X2046, Mmabatho 2735, South Africa

**Keywords:** *Bacillus*, beneficial microbes, bio-inoculants, in planta, maize-*fusarium* interaction, plant growth, *Pseudomonas*

## Abstract

Based on in vitro assessments, molecular and chemical analysis, *Pseudomonas fulva* PS9.1 and *Bacillus velezensis* NWUMFkBS10.5 are candidate biocontrol agents for plant disease management including maize fusariosis, a disease caused by members of the *Fusarium* species. This in vivo study evaluated the bio-protective potential of the aforementioned rhizobacteria strains on maize against the proliferation of the pathogenic fungus *Fusarium graminearum* (*Fg*). The study results show that the bacterized plants were not susceptible to *Fg* aggression and the antagonists displayed the capability to proliferate in the presence of other likely competing microflora. The screen-house data also suggest that the presence of resident soil microbiota impacted the activity of antagonists (PS9.1 and NWUMFkBS10.5). This variation was recorded in the soil treatments (sterilized and unsterilized soil). In all the experimental periods, bacterized maize plants with or without *Fg* inoculation significantly (*p* = 0.05) grew better in unsterilized soil. Besides, during the experimental periods, all the consortia treatments with or without *Fg* infection regardless of the soil used demonstrated appreciable performance. The result of this study suggests that the microbial agents can actively colonize the surface of their maize plant host, improve plant growth, and suppress the growth of phytopathogens. Considering their overall performance in this screen-house evaluation, *P*. *fulva* PS9.1 and *B*. *velezensis* NWUMFkBS10.5 have potential for field applications. All safety issues regarding their use under field conditions and risks associated with their extended-release into the environmental will, however, be assessed prior to further bioformulation, field investigation, and scale-up.

## 1. Introduction

Microflora that inhabit or colonize the rhizosphere can be classified based on the effects they have on plants, and plant roots can serve as a portal of entry for both beneficial and pathogenic micro-organisms that influence plant growth and development. This zone where root activity influences the biological interaction taking place between plant, soil, and resident flora significantly is referred to as the rhizosphere [1]. The influence of the beneficial micro-organisms present in the root zone on plant growth has been investigated for decades and these beneficial influences are exerted through direct and indirect mechanisms. The activities of these rhizobacteria could either result in the stimulation of plant growth or protection of the plant against pathogen attack [2,3,4]. Plant growth promotion involves the direct secretion of plant growth-regulators such as auxin, while biocontrol involves the production of metabolites such as siderophores, antibiotics, and hydrogen cyanide, respectively [5,6].

The large scale production of maize (*Zea mays* L.), which is one of the most important cereal crops cultivated globally, is adversely affected by members of the *Fusarium graminearum* species complex (FSGC) of which *F*. *graminearum* (*Fg*) has been frequently implicated in several worldwide outbreaks [7]. Infection of maize by this phytopathogen brings about devastating effects on plant parts, grain quality, and yield, resulting in significant economic losses in South Africa where maize is a major staple crop, used by humans for various industrial and agricultural purposes [8,9,10]. The occurrence of *Fg* in maize fields can be detected at both pre-harvest and post-harvest, and its infection occurs through several routes such as systemic infection through the seeds and the movement from the roots to the stalk, sometimes leading to severe rot of the whole plant [11]. Maize infection by *Fg* can also be via the silk channel, or through kernel and tassel injuries inflicted by insects or birds [12,13]. Furthermore, the mycotoxins (zearalenone, deoxynivalenol (DON)) produced by *Fg* when present in maize and maize-based products pose a health threat to man and their animals [10,14,15].

The biological control of this notorious plant pathogen gained worldwide attention due to the outbreaks of the FGSC in different geographic regions of the world [16,17]. The continued global awareness for less dependence on inorganic crops, chemical fertilizers, and agricultural activities that adversely affect the ecosystem, has also encouraged the introduction of biological disease management practices [1]. To date, few reports on effective strategies for biocontrolling *Fg* exist and the majority have not shown the effectiveness of indigenously developed biocontrol candidates in the control of localized maize fusariosis. The effectiveness of BCAs during in-planta applications is dependent on several environmental factors. Depending on the inherent conditions during pre-harvest or post-harvest of crops, seedling storage conditions, crop diseases, or phytopathogens intended to suppress (e.g., endophytes), bioprotectants can either be applied by drenching and coating seeds, or spraying of plant parts [18,19,20].

In previous reports, both the cells and crude extracts of *Bacillus velezensis* NWUMFkBS10.5 (Gram-positive) [21], and *Pseudomonas fulva* HARBPS9.1 (Gram-negative) [22], exerted biosuppressive activity against *Fg*. Here we assess the influence of these candidate biocontrollers on maize plants grown in the presence of the *Fg* pathogen during the maize pot experiment. The use of biological control consortia has been reported to be more effective during controlled bioprotection bioassays than the application of a single biological control agent [23]. In addition, biocontrol consortia strains have demonstrated their compatibility and synergy when applied in planta [24]. As such, this study will further evaluate the compatibility of test antagonists and consortia viability of the antagonists during the maize plant-*Fg* interaction in comparison to singularly applied antagonists. The study did not seek to evaluate disease incidence and severity.

## 2. Results

### 2.1. Compatibility of Test Antagonists, Seed Germination Test, and Seed Treatment Preparations

In the compatibility tests, there was no area of inhibition between the two test antagonist-bacteria (PS9.1 and NWUMFkBS10.5). Consequently, the test antagonist-bacteria were considered compatible with one another. During the seed germination test, all the seedlings tested sprouted healthily. Two hundred seeds were grown and they all sprouted. Growth parameters were, however, not recorded for the seed germination test (Figure S1). All the screen-house treatment combinations and soils used (sterile and unsterile) are shown in Table 1.

### 2.2. Harvest of Screen-House Pot Experiments Conducted over Three Experimental Periods

**Figure 1:**
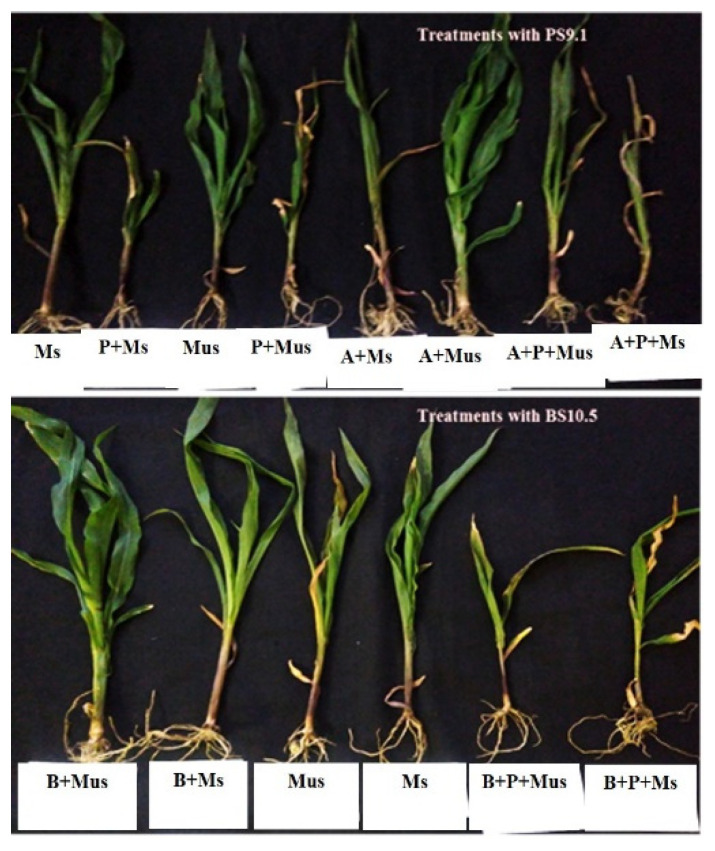
Harvest of plantings at V4-V5 stage (2.5 weeks) after seeding.

For the first experiment harvested at the V4-V5 stage, although plant growth was significantly (*p* = 0.05) retarded in the maize pots treated with only *Fg* spores and roots lacked vigor (Ms + P; Mus + P), no rot or wilting was observed (Figure 1; Table 2). In all the pots with non-bacterized seeds (Ms + P; Mus + P; Ms; Mus), the primary and lateral roots were not fibrous despite watering and the mesocotyl were unhealthy. Overall, better plant growth and pathogen suppression were recorded in the unsterilized soil. Although maize pots having treatments with NWUMFkBS10.5 (B + Ms; B + Mus) had better shoot length both in sterile and unsterilized soil, the fresh plant weight of the untreated plants (Ms; Mus) was significantly (*p* = 0.05) higher than all the other treatments (Table 2). Apart from the maize pots treated with only *Fg* spores, the consortia treatments with *Fg* spores (AB + P + Mus; AB + P + Ms) had the lowest shoot length. In addition, consortia treatments (AB + Mus; AB + Ms) had longer roots than all the other treatments and, from visual observation, plants treated with PS9.1 had more fibrous roots and root hairs (Figure 1).

**Figure 2:**
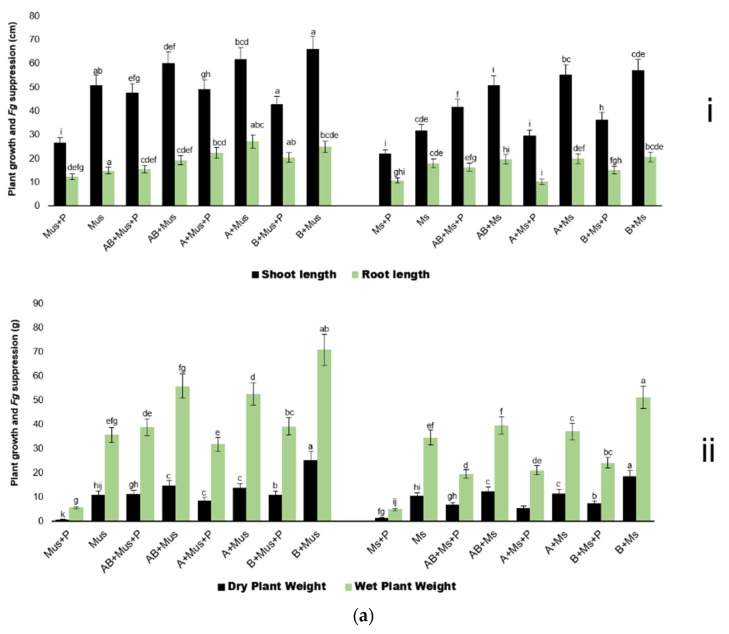

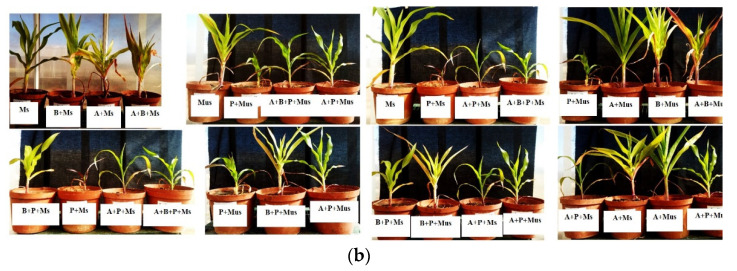
(**a**) (**i**,**ii**): Harvest of the second experiment at V6-V7 germination stage. All values are the means of three replicated pots from two repeats—presented as the mean ± SEM. Treatments are significantly different at *p* = 0.05 according to Duncan’s Multiple Range Test. (**b**): Harvest of the second experiment at V6-V7 germination stage.

The harvest of the second experiment showed that overall, the bacterized seed performed better in unsterilized soil; treatments in unsterilized soil without *Fg* showed a higher increase (*p* = 0.05) in plant vigor than plants in sterilized soil (Figure 2a(i,ii),b). The controls (maize pots treated with only *Fg* spores) had retarded growth as expected. Growth was also retarded in the non-bacterized maize seedlings in sterilized soil. 

Although antagonist NWUMFkBS10.5 treatments (B + Mus; B + Ms; B + Mus + P; B + Ms + P) performed significantly (*p* = 0.05) better than all other treatments in both soils, the consortia treatments (AB + Mus; AB + Ms; AB + Mus + P; AB + Ms + P) showed higher plant weights when compared with the PS9.1 treatments (A + Mus; A + Ms; A + Mus + P; A + Ms + P) (Figure 2a(i,ii),b). 

During the harvest of the third experimental period (Table 3), plant growth and fungal suppression after 90 days of infecting maize with *Fg* spores were recorded. Overall, all the treatments in unsterilized soil showed the best performance. 

**Figure 3:**
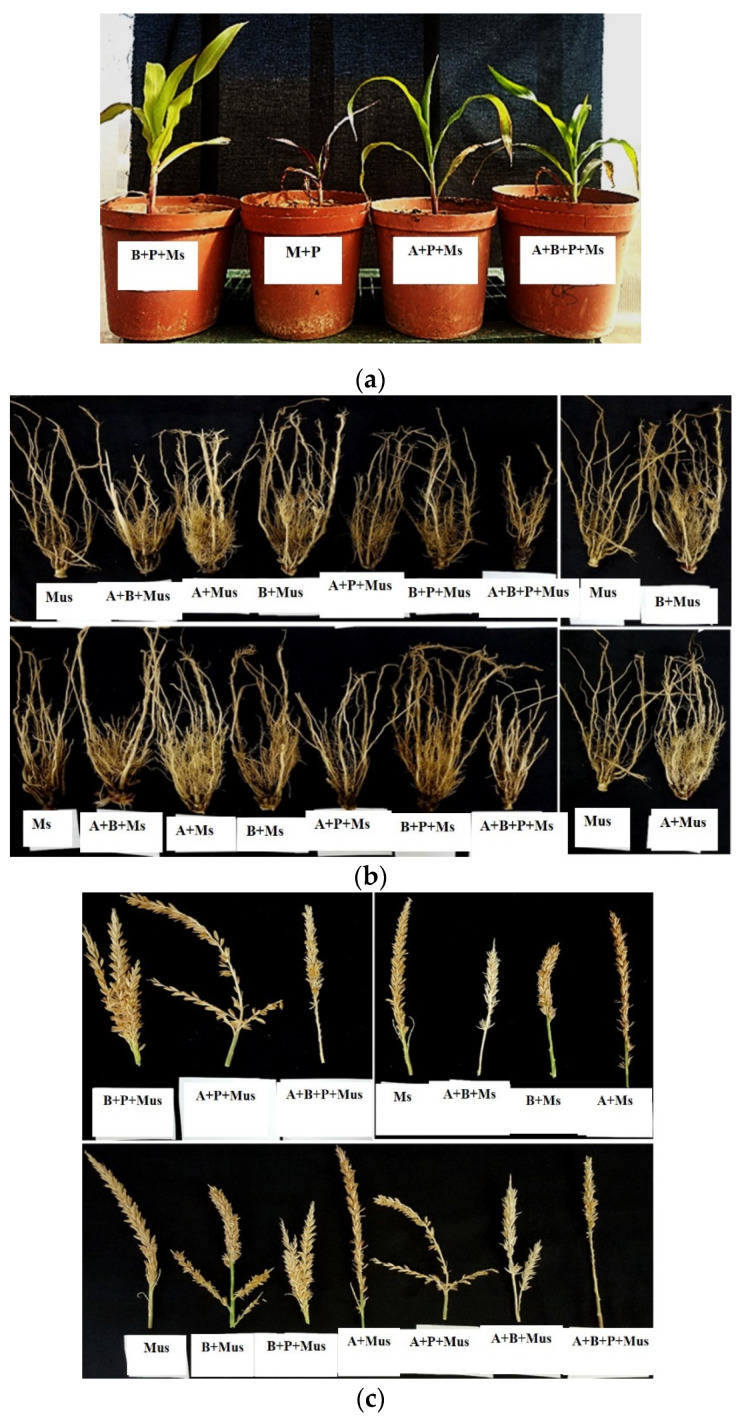
(**a**): *Fusarium graminearum* (*Fg*) aggression was observed in the non-bacterized maize seedling; (**b**): Bioprotective and growth-promoting effects of antagonists (*Pseudomonas fulva* PS9.1 and *Bacillus velezensis* NWUMFkBS10.5) seen on root system development; (**c**): Bioprotective and growth-promoting effects of antagonists (*Pseudomonas fulva* PS9.1 and *Bacillus velezensis* NWUMFkBS10.5) seen on tassel development.

NWUMFkBS10.5 treatments in unsterilized soil (B + Mus) without *Fg* inoculation, had the highest performance in all the parameters evaluated. The consortia (AB + Mus + P) with *Fg* inoculation had the best performance when compared with all other consortia treatments (B + Ms + P; B + Mus + P; A + Mus + P; A + Ms + P; AB + Ms + P) with *Fg* inoculation. As observed, the plants with only *Fg* treatments (Mus + P; Ms + P) without any bacterization dried and died off three weeks after seeding (Figure 3a). The primary and lateral roots were not fibrous despite watering in the pots with non-bacterized seeds (Figure 3b). The bioprotective effect of both antagonists on the root system and tassel development was, however, observed in other treatments (including seeds bacterized alone, seeds bacterized with *Fg*, and consortia) (Figure 3c).

## 3. Discussion

Management of grain fusariosis remains unalleviated and it might be necessary to integrate multiple plant disease approaches including efficient cultural practices, use of resistant cultivars for cultivation, or addition of a low concentration of fungicide or a combination along with the biocontrol agents to see how efficient they would be. A combination of several disease management practices has become the popular approach to managing the continued incidence of cereal grain fusariosis [25,26,27]. In this study, the influence of two candidate biocontrol rhizobacteria (*P*. *fulva* PS9.1 and *B*. *velezensis* NWUMFkBS10.5) on maize development in the presence of a phytopathogen was observed. However, to ascertain that the bacteria antagonist treatments were the only source of nutrients received by the plants during their germination period, no additional external fertilization or fungicide treatment was applied during the planting periods. Our results demonstrate that inoculation of the bacteria strains (PS9.1 and NWUMFkBS10.5) independently or in consortia did not only improve the maize plant growth under controlled conditions, but also ameliorated the detrimental effects of the *Fg* pathogen on the maize plant growth. This bioprotection against *Fg* aggression may be attributed to the antibiotic secreting potential of the bacteria antagonists or possible induction of systemic resistance in maize by the isolates [28,29]. This might be part of a further study to identify the responses elicited by maize as a result of treatments with these antagonists.

From the results, treatments in unsterilized soil with or without *Fg* pathogen showed a higher increase (*p* = 0.05) in plant vigor than plants in sterilized soil., This could be due to the influence or presence of transient microflora. Resident microflora has been implicated in plant growth promotion and biocontrol of phytopathogens in disease suppressive soils [30,31]. Although the antagonists exhibited compatibility in vitro, the consortia treatments (where *Fg* infection was excluded) had a slightly lower influence on plant growth and *Fg* suppression when compared to the singular bacteria mix treatments (B + Ms+; B + Mus; A + Ms; A + Mus + P/A + Mus + P). The consortia, however, enhanced plant growth and suppressed *Fg* activity better where *Fg* infection was included in the treatments. Whilst antagonist PS9.1 possesses some plant-growth-promoting and biocontrol compounds, rhamnolipids, pyoverdine, and rhizomide [22,32], the antagonist NWUMFkBS10.5 performed significantly (*p* = 0.05) better than PS9.1 in all the treatments. This could be attributed to the action of its multiple plant-growth-promoting and biocontrol biosynthetic compounds, macrolactin, bacillibactin, mersacidin, bacilysin, surfactin, difficidin, iturin, and fengycin [21,33]. A large number of the *Bacillus* and *Pseudomonas* spp. (e.g., *Bacillus velezensis* and *Pseudomonas putida*) harbor multiple beneficial genes in their genome [34] that confer on them better proliferative potential. The results of this study agree with previous studies seen under greenhouse and field trials in which species within the genera *Pseudomonas* and *Bacillus* suppressed the growth and aggression of *Fusarium* pathogens in cereal cultivars [35,36,37,38,39,40,41]

Despite the study not evaluating disease incidence and severity, the results of this investigation show that the bacterial treatments enhanced maize plant growth compared to the *Fg* inoculated controls and the untreated and non-bacterized (Mus; Ms) controls. In the non-bacterized *Fg* inoculated treatments, evidence of pathogen aggression was observed—there were no improvements in plant growth parameters in comparison to bacterized plants. Wilting was observed in the non-bacterized-*Fg* inoculated treatments—some of the plants died off (Figure 3a). The unhealthiness of the maize pots treated with only *Fg* spores (without any bacterization) could be attributed to *Fg* systemic infection [9]. The observable survival of the bacterized maize plants is not unexpected since they are no longer distressed, and they possess a non-diseased root system as shown in Figure 3b. This observation correlates with the report of Pandey, et al. [42]. Treatments with PS9.1 also had better root systems which were seen throughout all the pot experiments. We observed some discolorations in the tassels from the plants harvested from sterilized soils. Tassels from NWUMFkBS10.5 treated plants were significantly larger than the control and other treatments (Figure 3c). Besides, in the pot treatments with non-bacterized seeds, the primary and lateral roots were not fibrous despite watering and the mesocotyl were unhealthy.

Biocontrol inoculant formulations and their mode of delivery are important for their success under field conditions [1]. From the result we gathered, the root dip treatment of maize seedlings appears to be effective in conferring bioprotective ability on the maize. This correlates with the reports of Pal et al. [43] and Pereira et al. [44], in which pre-sowing of seedlings enhanced the activities of BCAs. The root dip treatment involves an inoculated release approach—microbial treatments influence plant development as they proliferate on and within plant parts. The root dip approach ensures that the microbial agents do not just actively colonize the surface of the host but may also become endophytic [45], possibly leading to the activation of the plant’s defense system. Although this study neither characterized soil microbiota nor evaluated bacteria persistence, the result from the unsterilized soil treatments suggests the possibility of transient soil microbiota playing a crucial role in the maize plant’s growth. Growth was significantly (*p* = 0.05) retarded in the non-bacterized maize seedlings in sterilized soil. Besides, the bioactivities of the antagonistic bacteria (*P*. *fulva* PS9.1 and *B*. *velezensis* NWUMFkBS10.5) in unsterilized soil are suggestive of their ability to persist and compete in the environment against resident microflora if they are to be considered as field biocontrol candidates. 

In recent years the biosafety status of candidate plant growth-promoting organisms (PGPO) and biocontrol strains have become a topic of major concern [46,47,48]. Despite strain PS9.1’s beneficial properties [22,32,49,50], some of its genetically related strains have also been recognized as opportunistic pathogens in previous studies [51,52,53,54,55,56]. Hence, PS9.1 can be considered a strain with overlapping biological impact, which poses a challenge for its use in planta. Because of the safety concerns regarding the release of candidate PGPO with possible overlapping (beneficial and deleterious) attributes [48], we recommend the cautionary use of strain *P*. *fulva* PS9.1. According to Keswani, et al. [48], strains of several bacteria genera exhibiting clinical and beneficial overlaps are still utilized in vitro as indicators of plant growth promotion and biocontrol, even though they are not commercially utilized because of the risk of pathogen outbreaks. We consider PS9.1 a prime candidate for the production of microbial synthesized bioinoculants without viable organisms since in silico genome analysis shows the bacterium also harbors biosynthetic genes for synthesis of pyoverdin, lankacidin C, and rhizomides (A, B, and C). Additionally, the candidate biocontroller (PS9.1) will likely be employed strictly for invitro investigative purposes under controlled environments. For example, studies involving the synthesis of agricultural metabolite additives lacking viable organisms will be conducted. We also intend to conduct further bioinformatic analysis of the PS9.1 sequenced genome [32], this should provide additional insights into its beneficial roles and limitations. Information gathered thereafter should be useful in evaluating the biotechnological importance of other beneficial plant-microbes with clinical overlaps.

## 4. Materials and Methods

### 4.1. Determination of Compatibility of Test Antagonists 

The bacteria antagonists used in this study, *P*. *fulva* PS9.1 and *B*. *velezensis* NWUMFkBS10.5, were previously isolated from the maize rhizosphere, identified based on the 16S rDNA gene sequence analysis (accession numbers MF098600 and KX353617.1), and analyzed by genome sequencing [21,22,32,33]. The *Fg* pathogen was provided as a gift. Bacterial cultures were preserved in 15% glycerol at –70 °C, Luria Bertani (LB) broth (Sigma Aldrich L3522) at 4 °C, and maintained on LB agar (Sigma Aldrich L3147) at 4 °C until needed. *Fg* was maintained on potato dextrose agar (PDA) (Sigma Aldrich P2182) at 4 °C until needed.

Before the pot cultivation (treatment of planting seeds and seeding), the compatibility of the rhizobacteria antagonists (PS9.1 and NWUMFkBS10.5) were determined through a dual-culture interaction test described previously by Dubey et al. [57] and Kumar, et al. [24], with slight modifications. An overnight pure culture of each test antagonist was subcultured on fresh LB agar plates. One hundred microliters of the suspension of each antagonist (OD_600 nm_ = 0.5) were then prepared and spot inoculated or streaked opposite each other on a LB agar for 48 h at 30 °C. Additionally, the PS9.1 antagonist was streaked on an overnight LB spread plate of antagonist NWUMFkBS10.5 and vice versa. The assay was incubated for 48 h at 30 °C. After incubation, plates were observed for overlapped growth of the antagonists and possible inhibition zones. The absence of inhibition zones and overlapping of growth indicated compatibility of the test antagonists while the presence of inhibition zone (if applicable) signified incompatibility.

### 4.2. Surface Sterilization of Maize Seeds and Seed Germination Test

Maize seeds variety DKC 73–72 (200 g) used in this study were obtained from NWK Limited (Econobuild) Mafikeng industrial area, North West Province, South Africa. To ensure the removal of fungicides from the seeds, they were washed in sterile distilled water. The seeds were further soaked in 0.75% Sodium hypochlorite solution for 5 min, followed by rinsing five times with sterile distilled water, and a final soaking on the fifth wash. The fifth wash was then inoculated on sterile nutrient agar plates to determine the sterilization efficiency. The surface disinfection was recorded as the absence of a colony-forming unit on the Nutrient agar (NA) plate (Millipore 70116).

The disinfected maize seeds were then subjected to a seed germination test based on the paper towel method (ISTA 2003) while the percentage germination (%) was calculated according to Abdul-Baki and Anderson [58]. One hundred maize seeds already lined with a paper towel (moistened with 10 mL of sterile distilled water) were arranged in three 1 L beakers (11.8 cm of side). The seeds were again covered with a paper towel (earlier moistened with 10 mL of sterile distilled H_2_O). The beaker was covered and incubated at room temperature for 4 days. Thereafter, the number of germinated seeds per beaker was counted to ascertain the germination percentage using the formula:

GP%=NG×100∕TNS

where *GP* = germination percentage; *NG* = Number of Germinated seed; *TNS* = total number of seeds. 

### 4.3. Screen-House Pot Experiment

#### 4.3.1. Collection of Soil for Pot Experiments

Planting soil was collected from the North-West University Animal Science Department agricultural planting area. Sterilization of a portion of the soil was done by dry heat for 1 week at 120 °C. Sterilized soil was plated on NA to confirm sterilization, and sterilization was continued until no growth was observed on the NA plates. Planting pots with dimensions 13 cm (diameter) × 10 cm (depth) were filled with 80 kg of both sterilized soil and unsterilized soil up to water-holding capacity. The sterile and unsterile soils were utilized to compare the persistence and competitiveness of the two rhizobacteria antagonists.

#### 4.3.2. Pre-Germination of Maize Grains for Pot Experiments

Two hundred grams of disinfected maize grains presoaked in sterile distilled water were placed in a 1 L beaker previously wallpapered with a sterile paper towel moistened with 10 mL of sterile distilled water. The seeds were covered again with the moistened (10 mL sterile distilled water) sterile paper towel. The flask was covered and incubated at 30 °C for 5 days after which only pre-germinated seeds with 2 cm callus were used for the seed-root dip pot experiment. 

#### 4.3.3. Seed Treatments Preparations

A modified seed-root dip pot experiment by Cook, Bruckart, Coulson, Goettel, Humber, Lumsden, Maddox, McManus, Moore, Meyer, Quimby, Stack and Vaughn [45] was employed for seed bacterization. From an overnight LB broth culture of PS9.1 and NWUMFkBS10.5, 20 μL of each isolate was transferred into 100 mL of LB broth in a 250 mL Erlenmeyer flask, and cultured for 3 days (28 °C) with continuous shaking at 150 g. Bacteria cells were recovered by centrifugation at 8000× *g* for 20 min and the supernatant was discarded. The pellet of each isolate was re-suspended in 100 mL sterile LB broth and optical density (OD) was adjusted to 0.5:600 nm. For single bacterization, 120 sterile pre-germinated maize seeds were submerged in the 100 mL bacteria inoculum (OD 0.5:600 nm) of each treatment, and the bacterized mixture consisted of 120 pre-germinated seeds submerged in 100 mL of co-inoculated bacteria broth (50:50 *v*/*v*) at OD 0.5:600 nm. This was incubated for 2–4 h with continuous shaking at 100 g for homogenization and adherence of bacteria to seeds. Sixty sterile pre-germinated maize seeds were also infected with 10^7^ spores mL^−1^ of the *Fg* pathogen. The overnight pre-germinated (120) bacterized seeds above were also air-dried and 60 grains were aseptically removed and submerged in the spore suspension of the *Fg* pathogen (10^7^ spores mL^−1^). All the treatments (Table 1) were incubated overnight to allow for adherence of inoculum to the seeds.

#### 4.3.4. Seed Cultivation and Planting Experimental Periods

A randomized complete block experimental design (with four replications per pot treatment) based on a modified protocol by Bacon and Hinton [59] was employed in this section. The germinated bacterized seeds, *Fg* inoculated seeds, and consortia were transferred to both soils (sterile and unsterile), with four seeds in one pot (pots in quadruplicate). Uninoculated seeds were used as a control and pots were watered with sterilized water. Furthermore, treatments consisting of 10 mL bacteria antagonist, 10 mL mixtures of both antagonists, and 10 mL of pathogen spore suspensions (10^7^ spores mL^−1^) were applied to the plants using a sterile syringe after one week of seeding in pots according to the treatments listed above. Planting was conducted in three experimental periods. The first experiment to determine the bioprotective capability of PS9.1 and NWUMFkBS10.5 during maize germination lasted 2.5 weeks in January 2017 after which the plants were harvested at the V4−V5 stage. The second experimental period occurred after the second inoculation of plants with *Fg*, and was for 40 days during February 2017−March 2017 and the third experimental period was for 3 months July 2017−September 2017. However, during the third experimental period, treatments consisting of 10 mL bacteria antagonist, 10 mL mixtures of both antagonists, and 10 mL of pathogen spore suspensions (10^5^ spores mL^−1^) were applied (following the experimental design and treatments) after 1 week of seeding, using a sterile syringe. During the third experimental period, the plants were harvested at the tasseling stage (VT). At the end of each experimental period, harvested plants were evaluated for growth and survival and growth parameters were recorded. The parameters measured at harvest were wet plant weight, shoot length, root length, and dry plant weight.

### 4.4. Statistical Analysis

Multivariate general linear model, Duncan Multiple Range Test, and Tukey test (highest significant different test (HSD)) were used to analyze and compare observed treatment means, pathogen–antagonist relationship, treatment effects, and interactions in SPSS statistical software (version 22) at the significance level of 5%. Prism 9.1.0 was used for further data analysis and visualization.

## 5. Conclusions

Integrating biocontrol approaches into the current cereal disease management practices will provide novel alternatives (in form of biofungicides) for cereal farmers and the grain industry for the control of inherent *Fusarium* pathogens. The significant findings of the study are the in planta potential of the *P*. *fulva* PS9.1 and *B*. *velezensis* NWUMFkBS10.5 andtheir capability to bioprotect maize plants against *Fg* fusariosis and their effectiveness in unsterilized soil. Considering biosafety issues, strain NWUMFkBS10.5 is the preferred candidate for the development of a biofertilizer, biostimulant, and biofungicide product. In as much as the maize germination was protected against *Fg* aggression, further work to determine the disease severity, reduction of mycotoxin contamination, and level of nutrient uptake by treated seeds after application of the rhizobacteria duo will be required to ascertain the complete bioprotective potential of these rhizobacteria strains.

## Figures and Tables

**Table 1:** Pot experiment treatment combinations for soils used (sterile and unsterile).

Sterile (Ms)			Unsterile (Mus)		
A	B	P	A	B	P
−	−	−	−	−	−
+	−	−	+	−	−
−	+	−	−	+	−
−	−	+	−	−	+
+	+	−	+	+	−
+	−	+	+	−	+
−	+	+	−	+	+
+	+	+	+	+	+

15 treatment combinations: (Ms + P; A + Ms; A + Ms + P; B + Ms; B + Ms + P; AB + Ms + P; AB + Ms; Ms; Mus + P; A + Mus; A + Mus + P; B + Mus; B + Mus + P; AB + Mus + P; AB + Mus; Mus). Key: M = Maize; A = PS9.1 inoculant; B = NWUMFkBS10.5 inoculant; P = pathogen inoculant (*Fusarium graminearum*); Mus = maize plant in unsterilized soil; Ms = maize plant in sterilized soil.

**Table 2:** Influence of antagonistic isolates (*Pseudomonas fulva* PS9.1 and *Bacillus velezensis* NWUMFkBS10.5) applied independently or in consortium against *Fg* and their effect on maize seedling growth parameters in a screen-house pot experiment (harvest of plantings at V4-V5 stage (2.5 weeks) after seeding).

Treatments	Shoot Length (cm)	Root Length (cm)	Wet Plant Weight (g)	Dry Plant Weight (g)
B + P + Ms	30.5 ± 0.5 ^d^	10.9 ± 1.1 ^e *Fg*^	2.7 ± 0.3 ^ef^	0.4 ± 0.1 ^de^
B + P + Mus	32.6 ± 0.5 ^c^	12.8 ± 1.0 ^cd^	3.1 ± 0.2 ^e^	0.5 ± 0.0 ^c^
A + P + Ms	27.4 ± 2.3 ^e^	9.6 ± 1.5 ^ghi^	1.9 ± 0.3 *^Fg^*	0.3 ± 0.0 ^de^
A + P + Mus	30.6 ± 0.4 ^d^	11.7 ± 0.6 ^def^	2.1 ± 0.3 *^Fg^*	0.4 ± 0.0 ^d^
AB + P + Ms	24.3 ± 2.4 ^e^	11.6 ± 0.7 ^def^	2.0 ± 0.3 *^Fg^*	0.4 ± 0.0 ^de^
AB + P + Mus	28.9 ± 1.6 ^d^	13.7 ± 1.3 ^bc^	3.1 ± 0.3 *^Fg^*	0.5 ± 0.0 ^de^
AB + Ms	31.9 ± 1.2 ^h^	18.5 ± 0.4 ^ghi^	3.9 ± 0.2 ^d^	0.7 ± 0.0 ^c^
AB + Mus	35.4 ± 1.7 ^f^	19.8 ± 0.5 ^def^	4.3 ± 0.2 ^d^	0.8 ± 0.0 ^b^
Ms	32.4 ± 0.9 ^c^	11.7 ± 0.3 ^def^	6.3 ± 0.1 ^ab^	1.3 ± 0.0 ^f^
Mus	36.3 ± 1.2 ^d^	17.9 ± 0.3 *^Fg^* ^h^	6.8 ± 0.1 ^a^	1.5 ± 0.0 ^e^
B + Ms	36.1 ± 1.4 ^ab^	14.7 ± 1.6 ^b^	5.9 ± 0.2 ^b^	0.9 ± 0.0 ^bc^
B + Mus	39.6 ± 0.6 ^a^	18.6 ± 0.9 ^a^	6.3 ± 0.2 ^b^	1.1 ± 0.1 ^a^
A + Ms	30.0 ± 0.6 ^d^	17.9 ± 0.6 ^e *Fg*^	4.4 ± 0.1 ^cd^	0.7 ± 0.0 ^d^
A + Mus	36.0 ± 0.9 ^b^	18.7 ± 1.4 ^cde^	5.8 ± 0.1 ^c^	0.9 ± 0.0 ^bc^
M + Ps	17.2 ± 0.9 ^i^	8.1 ± 1.2 ^i^	0.9 ± 0.1 ^h^	0.2 ± 0.0 ^g^
M + Pus	23.6 ± 0.6 ^g^	9.8 ± 0.8 ^hi^	1.7 ± 0.1 ^gh^	0.3 ± 0.0 *^Fg^*

Data represent means of three replicated pots from two repeats—presented as the mean ± standard error of mean (SEM). Columns with the same letter are not significantly different according to Duncan’s Multiple Range Test (*p* = 0.05) at each time of evaluation. Values with different uppercase letters are significantly different.

**Table 3:** Influence of antagonistic isolates (*Pseudomonas fulva* PS9.1 and *Bacillus velezensis* NWUMFkBS10.5) applied independently or in consortium against *Fg* and their effect on maize seedling growth parameters (harvest of third screen-house pot experiment).

Treatment	Shoot Length (cm)	Root Length (cm)	Fresh Shoot Weight (g)	Dry Shoot Weight (g)	Fresh Root Weight (g)	Dry Root Weight (g)
B + P + Ms	41.1 ^hi^	17.3 ^ef^	17.3 ^g^	4.4 ^g^	12.2 ^f^	1.8 ^fg^
B + P + Mus	35.7 ^i^	14.4 ^f^	16.1 ^g^	4.4 ^g^	11.6 ^f^	1.6 ^g^
A + P + Ms	45.1 ^gh^	18.3 ^ef^	29.6 ^e^	8.9 ^f^	15.1 ^e^	2.8 ^ef^
A + P + Mus	51.7 ^ef^	21.8 ^de^	36.4 ^d^	11.3 ^d^	18.5 ^cd^	4.1 ^d^
AB + P + Ms	44.4 ^gh^	17.9 ^ef^	24.6 ^f^	8.6 ^f^	15.0 ^e^	2.5 ^efg^
AB + P + Mu	50.0 ^fg^	19.1 ^e^	31.8 ^e^	9.1 ^ef^	16.3 ^de^	2.9 ^e^
AB + Ms	50.9 ^efg^	21.7 ^de^	36.3 ^d^	10.4 ^de^	16.5 ^de^	4.1 ^d^
AB + Mus	56.6 ^de^	24.8 ^cd^	39.5 ^cd^	11.4 ^d^	19.0 ^bc^	4.9 ^cd^
Ms	61.1 ^d^	24.9 ^cd^	39.6 ^cd^	11.5 ^d^	19.3 ^bc^	5.0 ^cd^
Mus	69.1 ^c^	26.2 ^cd^	40.5 ^cd^	14.2 ^c^	20.1 ^bc^	5.1 ^cd^
B + Mus	72.8 ^bc^	26.2 ^cd^	42.2 ^bc^	15.3 ^bc^	20.3 ^bc^	5.3 ^c^
B + Ms	78.8 ^ab^	32.6 ^b^	52.7 ^a^	18.2 ^a^	21.5 ^ab^	6.4 ^b^
A + Mus	74.2 ^bc^	29.2 ^bc^	45.2 ^b^	16.1 ^b^	21.3 ^ab^	6.3 ^b^
A + Ms	83.1 ^a^	38.5 ^a^	53.8 ^a^	18.3 ^a^	22.8 ^a^	7.4 ^a^
Mean SEM	58.2 0.6	23.8 0.4	36.1 0.37	11.6 0.1	17.8 0.22	4.3 0.1

All values are the means of four replicated pots–with overall mean ± SEM. Treatments are significantly different at *p* = 0.05 according to Duncan’s multiple range test. Values with same letters are not significantly different.

## Data Availability

Data sharing not applicable.

## Supplementary Materials

The following supporting information can be downloaded at: https://www.mdpi.com/article/10.3390/plants11030324/s1, Figure S1: Germination of maize seedlings prior to in vitro and in vivo usage: pre-germinated seeds submerged in the 100 mL bacteria inoculum (OD 0.5:600 nm) of each treatment.
