# Supplementary material for: Evaluation of Pseudomonas fulva PS9.1 and Bacillus velezensis NWUMFkBS10.5 as Candidate Plant Growth Promoters during Maize-Fusarium Interaction
Source: Plants (Basel). 2022 Jan 26;11(3):324. doi: 10.3390/plants11030324 (PMC8839840; doi:10.3390/plants11030324)
Supplement: Supplementary file 1 [file plants-11-00324-s001.zip › plants-1536121-supplementary.pdf]

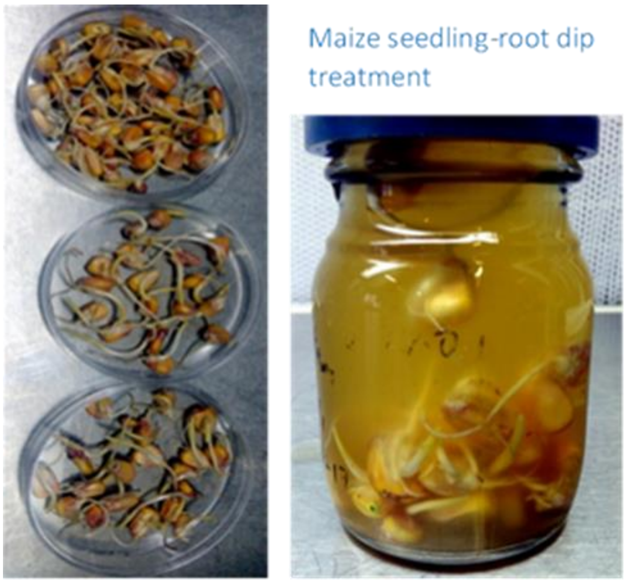

Figure S1. Germination of maize seedlings prior to in vitro and in vivo usage: pre-germinated seeds submerged in the 100 ml bacteria inoculum (OD 0.5:600 nm) of each treatment.

$$\% \; GP = NG \times 100 \; / \; TNS; \; \% \text{ GP} = 200 \text{ x } 100/200$$
